# Personal experience: Suicide and psychiatric care – a lament

**DOI:** 10.1192/pb.bp.113.046466

**Published:** 2015-02

**Authors:** Jeremy Holmes

**Affiliations:** 1 University of Exeter

## Abstract

A personal bereavement from suicide prompts a critique of current mental healthcare. Fragmentation, lack of long-term attachment to a tenured professional, the dearth of family therapy, and professional ambivalence are identified as weaknesses in current provision. Implicit is the case for change in UK psychiatric services, both structural (need for long-term therapies) and cultural (need for a mentalising rather than protocol-driven, ‘choice’-led ethos).

Recently, a close relation of mine died, through suicide. An intelligent, beautiful, creative young woman, for nearly 20 years she had suffered from bipolar disorder, for which she had received intermittent psychiatric care. Although the scale of my grief is minuscule compared with that of her immediate family, the poignancy was magnified, because I am, or was, a psychiatrist.

Inevitably, as one vainly tries to elude the irreversibility of time and loss, I have replayed in my mind the story of her illness and last days. Inescapably, too, I have felt on her behalf and that of her family a degree of guilt, anger and regret. Surely things could have been done differently, I think to myself. I look back on the patients with bipolar disorder I have treated over the years – those who have survived, and those unforgettable few, who, like my relative, took their own lives. I think of lessons learned, and not learned.

Although many aspects of psychiatric services have undoubtedly changed for the better (e.g. patient empowerment, multidisciplinary teamwork), there are also ways in which current psychiatry lets down its patients, especially when suicidal. What follows is perhaps yet another ultimately futile, counterfactual ‘if only...’ narrative that typically haunts the bereaved. But for all that, it may contain some validity.

## Death and psychiatry

There can be few psychiatrists who have not at some point been faced with the death by suicide of a patient under their care.^1^ Although psychological autopsy and critical incident review are unexceptionable, hospital authorities react very differently to death by suicide as compared with other deaths. Compared with the acceptance of the inevitable mortality associated with chronic physical illness, when a patient with a psychiatric illness takes their own life there is invariably an undercurrent of blame.^2^ Bipolar disorder, major depressive disorder and schizophrenia have a 20% mortality rate,^3^ comparable with multiple sclerosis^4^ or many forms of cancer, but the reactions they evoke are very different. This is reflected in the way that death by suicide is classified and recorded. Bipolar disorder, schizophrenia or borderline personality disorder are not in themselves ‘causes’ of death to be determined by a coroner. Suicide is grouped with the other stigmatised ‘-cides’, alongside homicide and infanticide.

Changing classification might well be desirable, but stigma-reducing biomedical attributions of ‘illness’ will still fail to capture the essence of the psychological pain with which the survivors are inundated. It is hard to accept that we can be so helpless in the face of unconscious forces over which we appear to have little control. The challenge to our sense of omnipotence and sense of freedom is overwhelming. There must, it seems, be an explanation, a narrative – someone or something to blame.

## Changes in psychiatric care – and their consequences

Official enquiries into untoward deaths usually end up with bland banalities such as ‘poor communication’ and ‘failure of adequate risk assessment’.^5^ But these gloss over the negative consequences of the many changes that have overtaken psychiatry in the past few decades.

The first is fragmentation of care. In the UK an ill patient is likely to be ‘looked after’ by at least three different groups in the course of her illness: the in-patient team, the assertive outreach team, and the continuing care team, each staffed by different people with differing philosophies, skills and limitations. Each team will be keen to get their job done and then pass the patient on, leading to eventual discharge. There is rarely one single individual who holds the patient in mind through all the phases of their illness, in sickness and in health. No ‘risk assessment’ protocol can substitute for this intimate knowledge, built up over time, of patients’ unique vagaries, strengths, weaknesses, vulnerabilities and inner workings.

This role could be, but so often is not, occupied by a senior tenured clinician – consultant psychiatrist, nurse-specialist, psychotherapist or clinical psychologist, supplemented by other members of the team. For this to happen there would need to be a move from short-termism and quick-fix problem-only therapies to long-term care for chronic illness. This would entail recognition that, as in Germany, long-term therapy, despite its cost, is economically efficient and can be available as part of comprehensive universal healthcare.^6^

### Attachment theory: Refuge

Attachment theory provides a possible scientific underpinning for this perspective. The distressed – and what is suicide if not the ultimate manifestation of distress? – are psychobiologically driven to seek out a secure base in the hope of alleviating their mental pain.^7^ In the absence of a secure base an abyss of despair and terror gapes, to which the illusory comfort of death may appear to provide a modicum of comfort.

Secure attachment is based on sensitive and responsive knowledge of the care-seeker, backed by ‘allo-parents’^8^ who augment and temporarily substitute, but can never fully replace, the primary attachment figure. The prevailing ‘customer-provider’ ethos, postmodern suspicion of inequalities in power relationships, and an underlying cost reduction imperative, are used to justify the current model. There is scant acknowledgement that the idea of ‘choice’ makes little sense in the context of severe mental illness. A commercial-type ‘contract’ anticipates, and tries to shape, the consumer’s needs, but is essentially non-‘mentalising’.^9^ It does not take account of the uniqueness of attachment relationships or attend to the inner world of experience that drives external behaviours.

Understanding a person’s inner world is not a recipe for vague psychological theorising, but can be intensely practical. A mentalising parent is able to plan effectively, take account of her own states of mind, and make sensible guesses about what is going on in her child’s mind. Similarly, a primary ongoing psychiatric attachment figure offers not just support and therapy to her patient with bipolar disorder, but, based on a shared journey through the vicissitudes of illness, gauges the need for medication, and helps the patient regulate the basic parameters of life – sleeping, eating, working, relating.^10^

### Long-term care has benefits

Soon after my relation died I dreamt I was in charge of her care; ‘You are going to stay in the ward, sectioned if necessary, until you are really well, even if that means staying here for a year!’, I said in my dream narrative. In reality this could, and probably should, be no more than a dream. In-patient beds are vanishingly scarce; ward culture inimical to long-term care; sectioning a highly articulate and plausible patient increasingly problematic. The idea of a hospital as an asylum, of therapeutic communities in which people with mental illness live and learn together, seems little more than a nostalgic memory. But in a psychiatric world without walls, the need for long-term care based on enduring relationships becomes all the more important, not least because the developmental experiences of those who suffer from mental illness are typically characterised by disorganised and disrupted attachments.^11^ The current climate tends to reproduce and reinforce rather than mitigate these adverse developmental experiences.

### The importance of family therapy

Finally – whatever happened to family therapy? From an attachment perspective family members – parents, spouses, siblings – however stressed, posses a unique sensitivity to the inner world of their loved ones. They have a lifelong baseline of normality against which to judge the subtle signs of relapse. They are an indispensible resource in which indefatigable altruism, based on the care-giving dynamic, can be taken for granted. Mental health professionals have often not yet fully thrown off their own adolescent rebellion, and too easily slip into excluding or even blaming the family, in part no doubt as a way of coping with the stresses of working in the beleaguered field of psychiatry. One consequence of ‘community care’, so called, is that families are relatively unsupported in their struggle to help their mentally ill relation, or cast as the ‘cause’ of the problem, and kept in the dark about professional formulation and planning.

Faced with the huge trauma of mental illness, the world typically becomes split into good and bad. When patients were detained for longer periods in psychiatric units, its staff at times became the necessary ‘bad object’, Rey’s ‘stone Mother’,^12^ a paradoxically safe container for all that was painful and destructive about mental illness. Hope and recovery were associated with discharge and resuming the ongoing connections represented by friends and family. Today, without the secure base function of the hospital, the family itself is too easily scapegoated, while professionals take refuge behind ‘confidentiality’ as a rationale for excluding family members. Skilled family therapists are a rarity, despite robust evidence that family intervention prevents relapse in serious mental illness.^13^

## Carrying on

Of course none of this call – for an attachment perspective, for more long-term therapy, for reviving therapeutic communities, for training family therapists – can reverse the horror of the loss one iota. When someone dies, from whatever cause, especially if young, a web of meanings, hopes and connections is severed. Restoration of meaning entails a painful recapturing and reworking of the past. The totality of the patient’s being – strengths, delights, loves, achievements, as well as suffering and pain – has to be sought and re-found. A similar task faces today’s psychiatric profession – to value the past, mourn what is irrevocably lost, reclaim what can be salvaged. In suicide, echoing Tennyson, a lifelong mourner,^14^ ‘much is taken’, but ‘much still abides’. This lament is a plea, when faced with suicide, for psychiatrists, alongside patients and their families, to ‘strive, to seek, to find, and not to yield’ – to fashion, finance or fatalism.

## References

[1] Michel K Jobes D (eds) Building a Therapeutic Alliance with the Suicidal Patient. APA Books, 2010.

[2] McClelland L Reicher S Booth N A last defence: the negotiation of blame within suicide notes. J Com Appl Soc Psychology 2000; 10: 225–40.

[3] Ösby U Brandt L Correia N Ekbom A Sparén P Excess mortality in bipolar and unipolar disorder in Sweden. Arch Gen Psychiatry 2001; 58: 844–50. 11545667 10.1001/archpsyc.58.9.844

[4] Hirst C Swingler R Compston D Ben-Shlomo Y Robertson N Survival and cause of death in multiple sclerosis: a prospective population-based study. J Neurol Neruosurg Psychiatry 2008; 79: 1016–21. 10.1136/jnnp.2007.127332 18303108

[5] Appleby L Kapur N Shaw J Hunt I While D Flynn S National Confidential Inquiry into Suicide and Homicide by People with Mental Illness. Annual Report. University of Manchester, 2013.

[6] Leichsenring F Rabung S Effectiveness of long-term psychodynamic psychotherapy: a meta-analysis. JAMA 2008; 300: 1551–65. 18827212 10.1001/jama.300.13.1551

[7] Holmes J John Bowlby and Attachment Theory (2nd edn). Routledge, 2013.

[8] Blaffer Hrdy S Mother Nature: Maternal Instincts and How They Shape the Human Species. Basic Books, 1999.

[9] Allen J Mentalizing in the Development and Treatment of Attachment Trauma. Karnac Books, 2012.

[10] Frank E Treating Bipolar Disorder: A Clinician’s Guide to Interpersonal and Social Rhythm Therapy. Guilford Press, 2005.

[11] Fonagy P Luyten P A developmental, mentalization-based approach to the understanding and treatment of borderline personality disorder. Dev Psychopathol 2009; 21: 1355–81. 19825272 10.1017/S0954579409990198

[12] Rey JH Universals of Psychoanalysis in the Treatment of Psychotic and Borderline States. Free Association Books, 1994.

[13] Falloon I Family interventions for mental disorders: efficacy and effectiveness. World Psychiatry 2003; 2: 20–8. 16946881 PMC1525058

[14] Ricks C Alfred Lord Tennyson: Selected Poems. Penguin, 1997.

